# Targeting mutant p53 with arsenic trioxide: A preclinical study focusing on triple negative breast cancer

**DOI:** 10.1016/j.tranon.2024.102025

**Published:** 2024-06-12

**Authors:** Subhasree Rajaram, Naoise C. Synnott, John Crown, Stephen F. Madden, Michael J. Duffy

**Affiliations:** a UCD School of Medicine, Conway Institute of Biomolecular and Biomedical Research, University College Dublin, Dublin D04 V1W8, Ireland; b Department of Medical Oncology, St. Vincent's University Hospital, Dublin D04 T6F4, Ireland; c Data Science Centre, School of Population Health, RCSI University of Medicine and Health Sciences, Dublin D02 YN77, Ireland; d UCD Clinical Research Centre, St. Vincent's University Hospital, Dublin D04 T6F4, Ireland

**Keywords:** Mutant p53, ATO, Triple-negative breast cancer, Therapy, RNA-seq, APR-246

## Abstract

New treatments are urgently required for triple-negative breast cancer (TNBC). As *TP53* is mutated in approximately 80% of TNBC, it is theoretically an attractive target for new drugs for this disease. Arsenic trioxide (ATO), which is used to treat promyelocytic leukaemia, was recently shown to reactivate mutant p53 and restore wild-type functionality. The aim of this study was to evaluate ATO as a potential new treatment for TNBC. Using a panel of 20 cell lines, we found that TNBC cell lines were more sensitive to ATO than non-TNBC cell lines (*P* = 0.045). Consistent with its ability to reactivate mutant p53, ATO was a more potent inhibitor of proliferation in cell lines with mutant *TP53* than the wildtype *TP53* (*P* = 0.027). Direct evidence of mutant p53 reactivation was the induction of multiple wild-type p53 canonical target genes such as *CDKN1A, SLC7A11, BBC3, PMAIP1, SESN2, SRXN1* and *TXNRD1*. Our findings support the activation of mutant p53 by ATO and, furthermore, the possible repurposing of ATO to treat *TP53-*mutated TNBC.

## Highlights

• Arsenic trioxide (ATO) was recently shown to reactivate mutant p53 and restore wild-type functionality.
• ATO was a more potent inhibitor of the proliferation of mutant p53 cell lines than wild-type p53 cell lines.
• Triple-negative breast cancer (TNBC) cell lines were more sensitive to ATO than non-TNBC cell lines.
• ATO induced wild-type p53 canonical target genes such as *CDKN1A, SLC7A11, HMOX1, BBC3, PMAIP1, SESN2, SRXN1* and *TXNRD1*.
• Our findings support the activation of mutant p53 by ATO and, furthermore, the possible repurposing of ATO to treat *TP53-*mutated TNBC.

## Introduction

Triple-negative breast cancer (TNBC), defined by the absence of estrogen and progesterone receptors and normal HER-2 gene expression, is an aggressive sub-type of breast malignancy [1]. While there has been some progress in the immunotherapy of this variant [[2], [3], [4], [5], [6]], the perceived lack of molecular targets makes it a difficult-to-treat entity with a poorer prognosis than other subtypes of breast cancer.

Amongst the most prevalent genetic alterations in TNBC are mutations in the *TP53* gene, which encodes the p53 protein [[7], [8], [9], [10]]. Across different studies, *TP53* was found to be mutated in approximately 80% of samples [[7], [8], [9], [10]]. Furthermore, a recent study showed that the growth and survival of a mouse TNBC were dependent on the presence of mutant p53 [11]. These studies, when taken together, suggest that *TP53* is a driver gene for TNBC and, thus, a potential target for new treatments for this aggressive subform of breast cancer.

Although, historically, mutant p53 proved difficult to target, several new strategies have recently been reported to be active against this mutant protein [12,13]. One of the most promising of these involves the use of low molecular weight compounds that convert the mutant conformation of the protein to a form possessing wild-type properties. These properties include the ability to inhibit cell proliferation and to induce apoptosis [12,13]. The advantage of this strategy is that it should not only eliminate the oncogenic properties of the mutant protein but also restore its wild-type functions. Several such compounds have been identified, with a small number recently progressing to clinical trials [14]. As of now, however, none have been approved for cancer treatment.

Following a search for compounds that convert mutant p53 to a wild-type-like form, Chen et al. [15] identified arsenic trioxide (ATO) as a potent reactivator of mutant p53 containing structural mutations (mutations that change the conformation of p53). It had much less effect on p53 with contact mutations (mutations that reduce the binding of p53 to DNA).

ATO differs from other mutant p53 reactivating compounds in that it has been in clinical use for several years, especially in the treatment of acute promyelocytic leukemia (APL) [16]. Its pharmaceutical properties and safety profile are thus well established [16,17]. Although subjected to detailed studies in APL (reviewed in Refs. [16,17]), there is little published data on the potential efficacy of ATO in solid tumours, including breast cancer.

The aim of our study was to evaluate the *in vitro* anti-cancer efficacy of ATO in breast cancer cell lines, focusing, in particular, on triple-negative (TN) cell lines.

## Materials and methods

### Cell culture

The origin and culturing of the breast cancer lines used were as previously described [18,19]. The p53 mutational status of each cell line is described in Suppl. Table 1. Briefly, BT-20, JIMT-1 and CAL-51 cells were cultured in DMEM media (Biosciences- Gibco). Hs 578t was cultured in DMEM with 10 µg/ml insulin (Sigma-Merck). UACC-812 was cultured in Lebovitz L-15 media (Biosciences- Gibco), supplemented with 1500 g/l sodium bicarbonate (Sigma-Merck) and 20 ng/ml recombinant epidermal growth factor (EGF) (Peprotech). All the other cell lines were cultured in RPMI media (Biosciences-Gibco). All media were supplemented with 10% fetal bovine serum (FBS) (Biosciences- Gibco) and 1% penicillin/streptomycin (HyClone). MCF 10A cells were cultured in DMEM/F-12 (Biosciences- Gibco) supplemented with 5% horse serum ((Biosciences- Gibco), 1% penicillin/streptomycin, 0.5 mg/ml hydrocortisone (Sigma-Merck), 100 ng/ml cholera toxin (Sigma-Merck), 10 µg/ml insulin (Sigma-Merck), 20 ng/ml human epidermal growth factor (EGF) recombinant protein (Biosciences- Gibco). The cell lines were incubated at 37 °C, supplemented with 5% CO_2_ and passaged 1–2 times per week.

### Source of reagents

ATO was purchased from Alfa-Aesar and dissolved in 1 M NaOH. The cytotoxic drugs, doxorubicin hydrochloride and docetaxel, were purchased from Sigma-Merck. N-acetyl cysteine (NAC), used at 10 mM, was purchased from Sigma-Merck and dissolved in water. Primers for the RT-qPCR analysis were purchased from Eurofins Genomics. Sequences of the primers used are listed in Suppl. Table 2.

### Measurement of cell viability and apoptosis

Cell growth was assessed using 3-(4,5-dimethylthiazol-2-yl)−2,5-diphenyltetrazolium bromide (MTT) (Sigma-Merck) as previously described [[18], [19]]. For measuring apoptosis, cells were seeded in 12-well plates at a density of 1.5 × 10^5^ cells/well and incubated overnight. Cells were treated with varying concentrations of ATO for 48 h. After treatment, cells were harvested and stained with annexin-V and propidium iodide using the Annexin V-FITC Apoptosis Detection kit (Invitrogen) following the manufacturer's instructions. The extent of apoptosis was detected by FACS analysis using BD FACSCanto™.

### RNA sequencing

3 × 10^5^ cells/well of the cell lines, MCF-7, MDA-MB-468, and BT-549 were seeded into a 6-well plate. Cells were treated with 10 µM ATO for 8 h. The total RNA from 4 biological replicate samples was extracted using the RNeasy mini kit (Qiagen) following the manufacturer's instructions. DNA digestion was performed in solution using the RNase-Free DNase from Qiagen. RNA clean-up was performed using the RNeasy mini kit (Qiagen) following the manufacturer's instructions. RNA concentration and integrity were determined by the Nanodrop spectrophotometer (LabTech) and the RNA 6000 Nano kit on the Agilent Bioanalyzer 2100 (Agilent systems), respectively. Strand-specific cDNA library (polyA selected) was sequenced using the Illumina NovaSeq platform (2 × 150 bp paired-end run) at Eurofins Genomics, Germany.

### RNA-seq analysis

Quality control was conducted using FASTQC. Reads were aligned to the human genome, version hg38, using the sequence aligner subread [20] via the Bioconductor R package Rsubread. The data were normalised using the mean-variance modeling (voom) method [21]. Multi-dimensional scaling (MDS) from the Rsubread package was used to generate ordination plots. One replicate from the control MCF-7 and MDA-MB-468 and one replicate from the ATO-treated MCF-7 and MDA-MB-468 were excluded from the analysis as they did not cluster with the other replicates from their cell type (i.e., they were outliers). Differentially expressed genes (DEGs) of the ATO-treated cells versus the control cells were determined using the ebayes function of the R package, Limma [22]. All *P* values were adjusted using the Benjamini and Hochberg method [23]. An adjusted *P*-value of < 0.05 and a log2 fold change (LogFC) greater than ± 1.2 were considered significant. Gene ontology (GO) analysis was performed using the ClusterProfiler package in R [24]. A *P*-value of < 0.05 was considered significant. Protein-protein interaction (PPI) analysis of DEGs was based on the STRING database, which contains known and predicted PPIs [25]. We focused on the top 100 DEGs to establish the PPI network.

### Gene expression analysis

1.5 × 10^5^ cells/well of the cell lines, MCF-7, MDA-MB-468, and BT-549 were seeded in a 12-well plate. Cells were treated with the indicated concentrations of ATO for 8 h. RNA was isolated using the RNeasy mini kit (Qiagen). DNA was digested on-column using the RNase-Free DNase from Qiagen following the manufacturer's instructions. Five hundred ng of RNA was reverse transcribed using a high-capacity cDNA reverse transcription kit (Biosciences). qPCR was performed on cDNA with PowerUp SYBR green mix (Biosciences), 250 nM forward and reverse primers (Suppl. Table 2) and analysed in a Light cycler 480 instrument (Roche). The 2^−∆∆CT^ method was used to calculate fold changes in mRNA expression relative to the β-actin housekeeping gene.

### ROS measurement

Cells were seeded in a 6-well plate at 3 × 10^5^ cells/well density and incubated for 24 h. Cells were then treated with 10 µM ATO for 8 h or 24 h. The cells were then analysed for ROS production using the CellROX™ deep red flow cytometry assay kit (Invitrogen) following the manufacturer's instructions. As a positive control, cells were treated with 250 µM of tert‑butyl hydroperoxide (TBHP) for 1 h. The negative control involved pretreatment with 10 mM N-acetyl cysteine (NAC) for 1 h before TBHP treatment. After treatment, cells were stained with CellROX® detection reagent for 30 min. Cells were then trypsinised and resuspended in PBS. SYTOX® dead cell stain was added to the cell suspension and incubated for 15 min. ROS production was detected by FACS analysis using BD FACSCanto™.

### Statistical analysis

All experiment data were obtained from at least 3 independent experiments performed in triplicates and presented as means ± *S*.E.M. Statistical analysis was calculated using GraphPad Prism version 5.0 software. A *P*-value < 0.05 was considered statistically significant. The IC50 value was determined using GraphPad Prism. Combination index (CI) values for assessing enhancement using combined treatment were calculated using CalcuSyn software (Biosoft). CI values < 1 at 50% inhibition were used to indicate synergism [26].

## Results

### Effect of ATO on breast cancer cell line proliferation

**Fig. 1:**
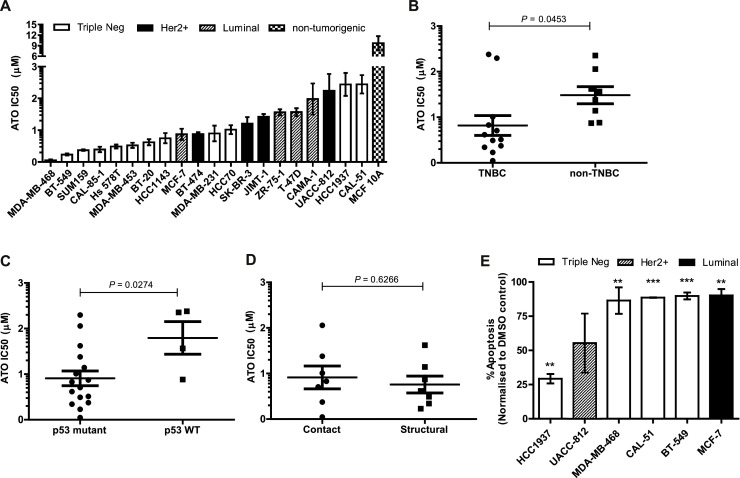
Effects of ATO treatment on proliferation and apoptosis of breast cancer cell lines. (A) Anti-proliferative effect of ATO was measured in a panel of 20 breast cancer cell lines and one non-tumorigenic immortalised breast epithelial cell line (MCF 10A) by the MTT assay. IC50 values were calculated and graphed using GraphPad Prism5. (B) Scatter-plot representation of the relationship between ATO IC50 values and triple-negative (TN) status of the cell lines, TNBC (*N* = 12) and non-TNBC (*N=* 8). (C) Scatter-plot representation of the relationship between the IC50 values for ATO in mutant *TP53 (N=16)* versus wild-type *TP53* cell lines (*N* = 4). (D) Scatter-plot representation of the relationship between IC50 values for ATP and p53 mutational subtype of the cell lines- contact mutation (*N=7*), structural mutation (*N* = 7). (E) Apoptosis induced following treatment with 10 µM ATO for 48 h in selected breast cancer lines and analysed by flow cytometry. Data are mean ± *S*.E.M of 3 independent experiments. *P < 0.05, **P < 0.01, ***P < 0.001 using Student's *t*-test analysis.

The effect of ATO on cell proliferation was investigated using the MTT assay in a panel of 20 breast cancer cell lines representing the 3 major molecular subtypes of breast cancer, luminal (*N* = 4), HER2-positive (*N* = 4) and triple-negative (TN) (*N* = 12). IC50 values of ATO for the inhibition of cell growth ranged from 50 nM to 2.4 µM (Fig. 1A). In contrast to the breast cancer cells, the IC50 value of ATO for the MCF-10A cell line, a non-tumorigenic immortalised breast epithelial cell line, was higher at 9.72 µM. Thus, although the proliferation of this non-malignant breast cancer was inhibited by ATO, the magnitude of inhibition tended to be less than that of all the malignant cell lines studied.

Of potential clinical importance was our finding that TN cell lines were more sensitive than non-TN cell lines, i.e., had significantly lower IC50 values (*P* = 0.045) (Fig. 1B). Furthermore, we found significantly lower IC50 values for cell lines expressing mutant p53 compared to cell lines having wild-type p53 (*P* = 0.027) (Fig. 1C). Although previously reported to be a more potent reactivator of p53 with a structural mutation than with contact mutations [15], we found that ATO exhibited similar anti-proliferative activity irrespective of the type of *TP53* mutation (Suppl. Table 1) present in the breast cancer cell lines (Fig. 1D).

It is important to state here that the concentrations of ATO that we used in the above *in vitro* experiments, i.e., 0 to 10 µM, are likely to be effective *in vivo* as the reported circulating doses of the drug when used in the treatment of APL are mostly between 0 and 20 µM [27].

### Effect of ATO on apoptosis

To identify potential mechanisms involved in growth inhibition, ATO-treated cells were analysed for apoptosis. Apoptosis was investigated in 2 cell lines with wild-type *TP53* (CAL-51, MCF-7), 2 mutant *TP53* TNBC lines with low IC50 values (i.e., < 0.3 µM) (MDA-MB-468, BT-549), and 2 mutant *TP53* lines with high IC50 values (i.e., > 2.3 µM) (HCC1937, UACC812). The level of apoptotic induction varied by cell line, with the sensitive lines (lower IC50 values) tending to have a higher percentage of apoptotic cells compared to the less sensitive cell lines (high IC50 values) (Fig. 1E). The wild-type *TP53* cell line, CAL-51 however, had a higher percentage of apoptotic induction (90%), despite having a relatively high IC50 value.

### Comparison of the anti-proliferative effects of ATO with Eprenetapopt (APR-246) and COTI-2

**Fig. 2:**
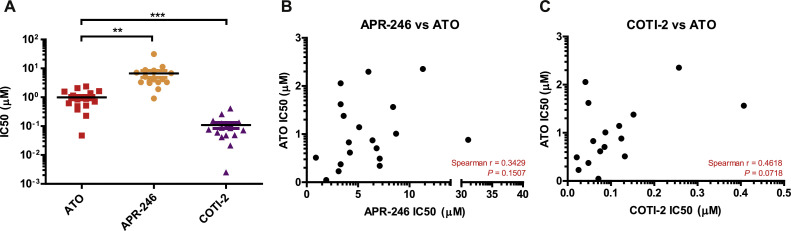
Relationship between IC50 values of ATO versus APR-246 or COTI-2. (A) Relative IC50 values for ATO, APR-246 and COTI-2. Data were analysed using Student's *t*-test in GraphPad Prism5. *P < 0.05, **P < 0.01, ***P < 0.001. (B) Scatter plot representation of the relationship between IC50 values for ATO and APR-246. (C) Scatter plot representation of the relationship between IC50 values for ATO and COTI-2. Data were analysed using two-tailed Spearman correlation in GraphPad Prism5.

Eprenetapopt (APR-246) and COTI-2 are 2 mutant p53 reactivator drugs that have progressed to evaluation in clinical trials. As these 2 compounds were previously investigated by us in breast cancer cells [18,28], we decided to compare their response with that of ATO. As shown in Fig. 2A, the IC50 values for ATO were significantly lower than for APR-246 but higher than for COTI-2. However, there was no significant correlation between the IC50 values for ATO and those for APR-246 (Fig. 2B) or COTI-2 (Fig. 2C).

### Effect of ATO in combination with cytotoxic compounds on breast cancer cell line proliferation

**Fig. 3:**
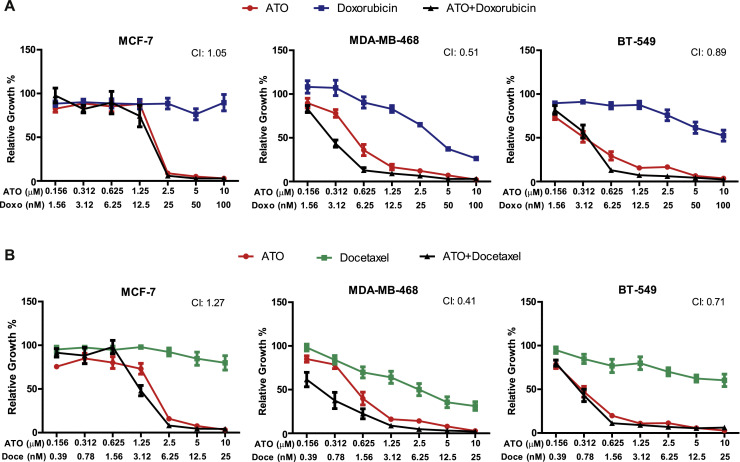
Effect of combined treatment with ATO and commonly used cytotoxic drugs on cell proliferation. Cells were treated with various concentrations of ATO in combination with (A) doxorubicin or (B) docetaxel for 5 days. Cell growth was assessed by the MTT assay. Combination indices (CI) values were calculated using the Compusyn software, as mentioned in the Materials and Methods section. CI < 1 indicates drug synergy. Data are mean ± *S*.E.M of 3 independent experiments.

To enhance the inhibition of cell proliferation, ATO was combined with the clinically used chemotherapeutic drugs for breast cancer, doxorubicin or docetaxel. In both the mutant *TP53* cell lines, MDA-MB-468 and BT-549, combined treatment with ATO and doxorubicin produced a synergistic effect on cell growth inhibition (CI < 1.0) (Fig. 3A). Similar results were found when ATO was combined with docetaxel (Fig. 3B). In contrast, no such synergism was observed with either drug combination in the wild-type *TP53* cell line MCF-7.

### Effect of ATO on global gene expression

To investigate the effect of ATO on global gene expression, we treated the wild-type *TP53* cell line, MCF-7 and 2 *TP53* mutant TNBC cell lines, MDA-MB-468 (contact mutation, p.Arg273His) and BT-549 (structural mutation, p.Arg249Ser) with either vehicle or ATO. We deliberatively selected a short exposure time of 8 h to detect early response genes to ATO treatment. Analysis by MDS following RNA-seq revealed that the ATO-treated samples were separated from the control sample (Suppl. Fig. 1A) (As mentioned in the Methods sections above, outliers were not included in the analysis).

**Fig. 4:**
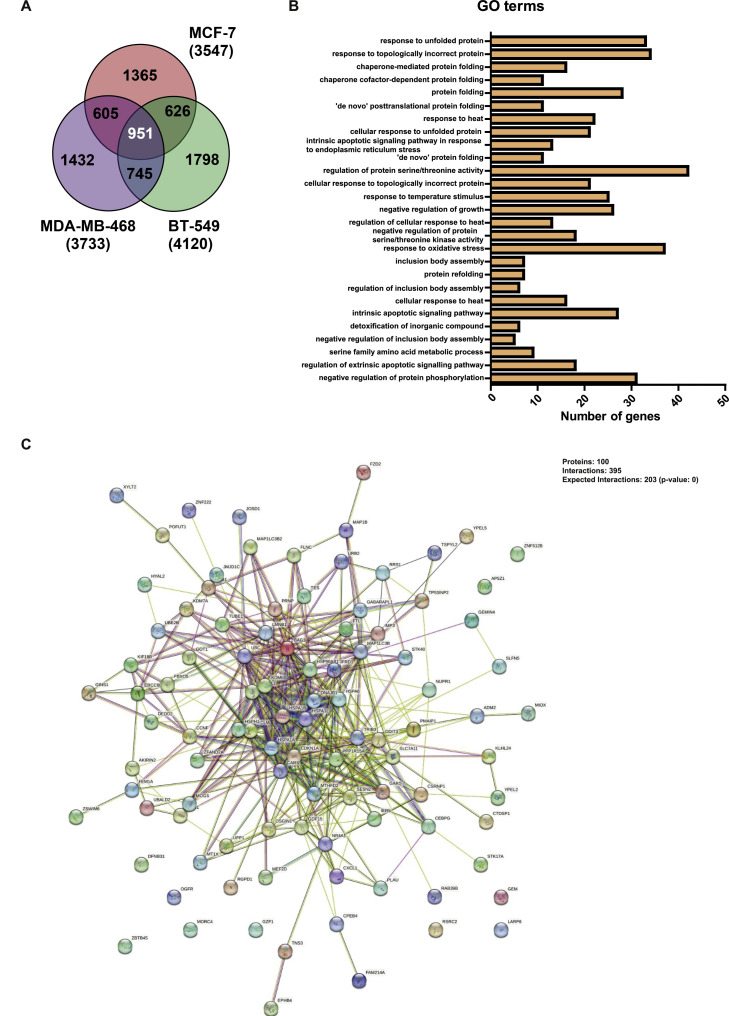
Functional enrichment analysis of RNA-seq data. MCF-7, MDA-MB-468 and BT-549 cells were treated with 1 M NaOH (control) or 10 µM of ATO for 8 h. (A) Venn diagram showing the overlap in DEGs across MCF-7, MDA-MB-468 and BT-549 cells following treatment with ATO. (B) Gene ontology terms enriched for the 951 overlapping genes across the 3 cell lines. (C) Protein-protein interaction (PPI) network analysis of the top 100 DEGs from the overlapping gene list.

A total of 3547 (MCF-7), 3733 (MDA-MB-468), and 4120 (BT-549) genes were differentially expressed between the ATO-treated and the control cell lines. Consistent with a previous report showing that wild-type p53 upregulated more genes than it downregulated [29], we show that treatment with ATO increased the expression of more genes than it decreased. Volcano plot distribution of the DEGs for each cell line is provided in Suppl. Fig. 1B. The top 25 differentially regulated genes for each cell line are listed in Suppl. Tables 3–5, while the full list of differentially regulated genes in each cell line is shown in Suppl excel files 1, 2 and 3. Comparisons of the DEGs between all 3 cell lines identified 951 overlapping genes (Fig. 4A). GO enrichment analysis on these 951 overlapping DEGs identified several genes involved in protein folding/protein unfolding responses, such as response to unfolded protein, response to topologically incorrect protein, and chaperone-mediated protein folding (Fig. 4B). Other frequently regulated processes identified genes involved with the response to heat, regulation of intrinsic apoptotic signaling, regulation of extrinsic apoptotic signaling, and regulation of protein serine/threonine kinases and response to oxidative stress.

To further investigate key DEGs involved in ATO action, the interactions of the top 100 overlapping DEGs underwent protein-protein interaction analysis [25]. We found that there was a total of 73 nodes and 790 interactions in the PPI network (Fig. 4C). We identified 13 proteins that had > 20 interactions in the network (Table 1). Approximately half of these top interacting proteins belong to the heat-shock family of proteins such as DNAJB1, HSPA6, BAG3, HSPA1A, HSP90AB1 and HSPH1. These proteins are potentially involved in regulating the stability of p53 [30].

**Table 1:** Genes with 20 or more interactions in the PPI network following treatment with ATO.

Accession number	Gene symbol	Number of interactions
ENSP00000318687	*HSPH1*	20
ENSP00000360609	*HSP90AB1*	20
ENSP00000253063	*SESN2*	24
ENSP00000261366	*LMNB1*	26
ENSP00000364802	*HSPA1A*	26
ENSP00000358081	*BAG3*	28
ENSP00000252809	*GDF15*	30
ENSP00000310219	*HSPA6*	30
ENSP00000268607	*MAP1LC3B*	32
ENSP00000254322	*DNAJB1*	42
ENSP00000254846	*KDM6B*	42
ENSP00000200453	*PPP1R15A*	46
ENSP00000217233	*TRIB3*	46

### Effects of ATO on the expression of p53 target-genes

**Fig. 5:**
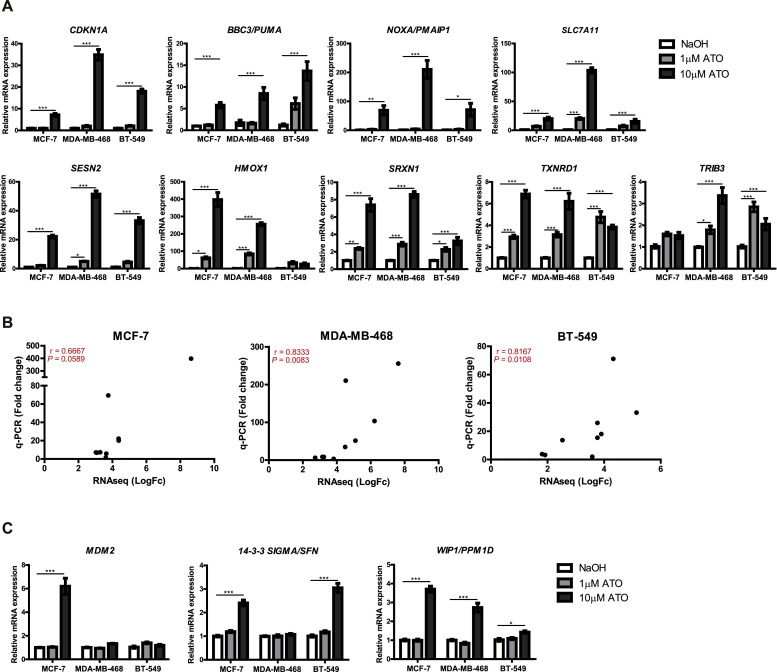
RT-qPCR analysis of ATO-regulated genes. (A) RT-qPCR validation of 9 p53-regulated genes in MCF-7, MDA-MB-468 and BT-549 cells using RNA sequencing (RNA-seq). Cells were treated with either control, NaOH, 1 µM ATO or 10 µM ATO for 8 h. (B) Correlation between expression levels of p53 regulated genes expression using RNA-seq and RT-qPCR in MCF-7, MDA-MB-468 and BT-549 cells. (C) Effect of ATO on expression of canonical p53 regulated genes not identified using RNA-seq. Data (A, C) are mean ± *S*.E.M of 3 independent experiments performed in triplicates. *P < 0.05, **P < 0.01, ***P < 0.001 using two-way ANOVA with Bonferroni post-test analysis.

To investigate the effect of ATO on mutant p53 reactivation, we compared our differentially expressed gene list to a high-confident p53 target-gene list published by Fischer [29]. We identified 104 (MCF-7), 90 (MDA-MB-468), and 101 (BT-549) classical p53 target-genes differentially regulated by ATO (Suppl. Fig. 2). Of these, 33 genes were differentially regulated in all the 3 cell lines (Suppl. Table 6). To further validate the RNA-seq results, we performed RT-qPCR on selected 9 canonical p53 target-genes, i.e., *CDKN1A, BBC3, PMAIP1, SLC7A11, SESN2, HMOX1, TRIB3, SRXN1, and TXNRD1* that were identified among the top 25 DEGs in all 3 cell lines and that were also present among the top 100 interacting proteins from the PPI network. Consistent with the RNA-seq data, the expression of 7 of the 9 canonical p53-regulated genes investigated was significantly increased in the 3 cell lines investigated (Fig. 5A). The 2 genes whose expressions were not increased in all the cell lines were *HMOX1* and *TRIB3*. Although *HMOX1* was significantly increased in MCF-7 and MDA-MB-468 cells, it was not elevated in the BT-549 cell line. As for *TRIB3,* its expression was increased in MDA-MB-468 and BT-549 cells but not in the MCF-7 cell line. Importantly, the increase in expression levels detected by RT-qPCR was highly correlated with that of RNA-seq, i.e., the correlation coefficients between RT-qPCR and RNA-seq results for MCF-7, MDA-MB-468 and BT-549 were 0.6667, 0.8333 and 0.8167, respectively (Fig. 5B).

### Effects of ATO on canonical p53 target-gene expression not detected using RNA-seq

Since genes reported to be classical p53 target genes [29], such as *MDM2, SFN* (14–3–3σ), and *PPM1D* (WIP1) were not present in our overlapping list from the RNA-seq analysis, we used RT-qPCR to investigate a possible effect of ATO on these genes (Fig. 5C). Treatment with 10 µM ATO increased the expression of *MDM2* in the wild-type *TP53* cell line, MCF-7, but not in the 2 cell lines expressing mutant *TP53*. ATO also increased the expression of *SFN* in MCF-7 and BT-549 but not in MDA-MB-468 cells. *PPM1D* was found to be induced in all 3 cell lines.

### Effect of ATO on induction of reactive oxygen species (ROS)

**Fig. 6:**
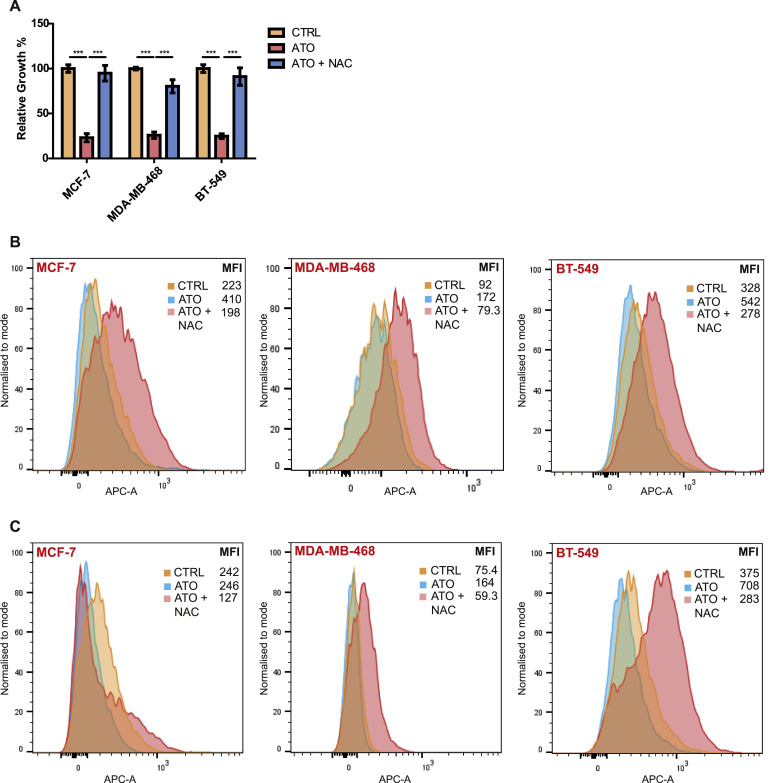
Effect of ATO on ROS production. Cells were treated with 10 µM ATO with +/- pretreatment with N-acetylcysteine (NAC) for 1 h. (A) Cell growth was measured by MTT assay after 48 h of treatment with ATO. Data are means ± *S*.E.M of 4 independent experiments in duplicates. Measurement of the level of ROS using a flow cytometer after (B) 8 h treatment and (C) 24 h treatment with ATO. Mean fluorescence intensity (MFI) was determined by FlowJo software. Data are representative of 4 independent experiments. *P < 0.05, **P < 0.01, ***P < 0.001 using two-way ANOVA with Bonferroni post-test analysis.

Mutant p53 reactivating drugs such as APR-246 have been shown to increase ROS formation [31,32]. To investigate if ATO acts similarly, we pretreated cells with the ROS-scavenging antioxidant N-acetyl cysteine (NAC). As shown in Fig. 6A, NAC pretreatment restored the growth of cells across all 3 cell lines. Using flow cytometric analysis, we showed that ATO increased ROS levels following 8 h of treatment (Fig. 6B). However, at 24 h, there was no increase in ROS levels in MCF-7 cells (Fig. 6C). In comparison, the increased level of ROS was sustained at 24 h in the two p53 mutant lines, MDA-MB-468 and BT-549 (Fig. 6C). Treatment with NAC reduced levels of the reactive oxygen species to concentrations similar to that in the control samples.

## Discussion

Our findings reported here are amongst the first to investigate, in detail, the effects of ATO on breast cancer cells. Consistent with its ability to reactivate mutant p53 [15], we show that ATO was more potent in inhibiting proliferation in mutant *TP53* cell lines than in wild-type *TP53* cell lines. A potentially important result from a clinical perspective was our finding that the anti-proliferative response to ATO was significantly greater in TN than in non-TN cell lines. This is potentially of great relevance given the relative paucity of clinically exploited molecular targets in this subtype of breast cancer. We have previously reported similar findings for other mutant p53 reactivating compounds, APR-246 and COTI-2 [18,28]. Our results also show that the anti-proliferative effect of ATO is higher on cancer cell lines compared to the non-tumorigenic cell line, MCF-10A. Consistent with our findings, Smith et al. [33] previously reported that high exposure to inorganic arsenic in drinking water resulted in a significant reduction in breast cancer mortality. These authors also found that ATO induced apoptosis and reduced viability in 4 breast cancer cell lines. However, in contrast to our work, the mechanism of action of ATO was not investigated in that study.

As mentioned above, Chen et al. [15] reported that ATO reactivated mutant forms p53 with certain types of structural mutations but had minimal impact on p53 with contact mutations. Our results, however, suggest similar anti-proliferative effects in breast cancer cell lines, irrespective of whether they had *TP53* contact or structural mutations. Furthermore, we found that ATO regulated p53 canonical genes in a cell line with a contact mutation (MDA-MB-468) as well as in a cell line with a structural *TP53* mutation (BT-549). These results suggest that ATO may have anti-proliferative and, thus, potential anticancer activity irrespective of the type of *TP53* mutation present. If confirmed, this would broaden the potential scope of ATO to treat p53 mutant cancers. Of course, it is possible that at least some of the antiproliferative effects of ATO are mediated independently of mutant p53 reactivation, which could potentially provide an explanation for the antiproliferative effects in cell lines with contact mutations.

A possible ATO effect induced independently of mutant p53 is the production of ROS. As was previously reported with the mutant p53 reactivating compounds, APR-246 and PK11007 [31,32,34], as well as several other anticancer drugs [35], we found that treatment with ATO also increased the formation of ROS. High concentration of ROS may at least partly contribute to the anticancer activity of ATO, as excess levels of the reactive species can induce oxidative stress, damage DNA, inactivate proteins and cause lipid peroxidation, which in turn can lead to cell death [36,37]. Indeed, cancer cells are believed to be more sensitive than normal cells to high levels of ROS [36,37]. This ability to increase ROS levels may enable ATO to exert anti-proliferative/proapoptotic effects independent of mutant p53 reactivation and consequently enhance its anti-cancer activity.

While some of the anti-cancer activity of ATO may be caused *via* ROS induction, our data using RNA-seq and RT-PCR are also consistent with ATO-mediated mutant p53 reactivation to a wild-type form. In particular, we showed that ATO increased the expression of multiple canonical wild-type p53-regulated genes such as *CDKN1A, SLC7A11, BBC3* (Puma) and *PMAIP1* (Noxa) across the 3 cell lines investigated. Interestingly, all these genes were previously reported to be upregulated by APR-246 [38]. ATO, however, failed to increase the expression of the established wild-type p53-regulated gene *MDM2* in the *2* mutant p53 cell lines. In apparent contrast to our finding, Song et al. [39] reported that ATO upregulated *MDM2* in cell lines with so-called efficiently rescued type 1 *TP53* mutations. This apparently conflicting finding might be explained by our relatively short incubation period with ATO, i.e., 8 h, compared to the 24 h incubation period used by Song et al. [39]. Alternatively, the different findings may relate to the effects of ATO being cell line [40] or *TP53* mutation-specific [39].

In addition to upregulating several established p53-regulated genes, we show that treatment with ATO altered pathways that are consistent with p53 refolding and, consequently, reactivation. Thus, using GO enrichment analysis, most of the top 10 ranked terms were related to protein folding/unfolding, while another highly ranked term, i.e., the extrinsic apoptotic signaling pathway, was previously reported to be one of the most highly ranked p53 regulated pathways [29].

It could be argued that our work requires confirmation in animal models prior to any clinical trial. ATO has, however, been in clinical use for several years [15,16], and its pharmacodynamics and toxicological properties are well established. However, like all systemic anti-cancer therapies, ATO has side effects such as inducing the differentiating syndrome, causing liver toxicity and promoting cardiac conduction abnormalities [41]. Despite these toxicities, ATO, in combination with all-trans retinoic acid, is the standard treatment for patients with APL and, importantly, avoids the use of chemotherapy in this disease. Thus, because of its extensive investigation and manageable toxicity, we believe that additional toxicity studies in animals are not strictly necessary. Indeed, while this manuscript was in preparation, Li et al. [42] reported that administration of ATO significantly increased the survival of Li-Fraumeni syndrome mimicking mice without apparently causing major toxicity. (Li-Fraumeni is a hereditary syndrome involving mutations in one allele of TP53, which give rise to multiple types of cancer, including breast cancer).

In summary, we show that ATO has anti-proliferative and pro-apoptotic activity in breast cancer cell lines. Furthermore, ATO exhibited greater anti-proliferative efficacy in TN than in non-TN cell lines and in mutant *TP53* cells than in wild-type *TP53* cell lines. Overall, our data suggests that at least some of the anti-proliferative effects of ATO are due to the rescue of mutant p53, although we cannot exclude the possibility that ATO has activity independent of mutant p53 reactivation. Based on our findings, we suggest that ATO should now be considered for investigation in a clinical trial in patients with mutant *TP53* TNBC. Such a trial should include the measurement of the *TP53* mutation type of the tumor being treated. Co-incidentally, ATO is currently undergoing multiple clinical trials in patients with a variety of different types of *TP53* mutated cancers, including ovarian cancer (ClinicalTrials.gov Identifier, NCT04489706), endometrial cancer (ClinicalTrials.gov Identifier, NCT04489706), a basket trial in patients with diverse refractory solid tumours (NCT04869475) as well as in patients with various types of leukemia (ClinicalTrials.gov Identifier, NCT03381781; ClinicalTrials.gov Identifier: NCT03377725). TNBC, which still lacks an effective targeted therapy, might now be added to that list.

## Data availability

Sequencing data is made available on the SRA (BioProject: PRJNA1101845). All other data are available from the authors on request.

## Funding information

This work was supported by the Cancer Clinical Research Trust, Dublin, Ireland.

## CRediT authorship contribution statement

**Subhasree Rajaram:** Writing – original draft, Visualization, Validation, Investigation, Formal analysis. **Naoise C. Synnott:** Validation, Investigation, Formal analysis. **John Crown:** Writing – review & editing, Supervision, Funding acquisition. **Stephen F. Madden:** Writing – original draft, Visualization, Supervision, Software, Formal analysis. **Michael J. Duffy:** Writing – review & editing, Writing – original draft, Supervision, Project administration, Funding acquisition, Conceptualization.

## Declaration of competing interest

The authors declare the following financial interests/personal relationships which may be considered as potential competing interests: SR, NCS, SM and MJD declare no conflict of interest. JC: Research funding (to institution): Eisai, Puma Biotechnology, Roche, Boehringer Ingelheim; Employment: OncoMark, Ltd.; Honoraria: Eisai, Puma Biotechnology; MSD Oncology, Pfizer, G1 Therapeutics; Novartis; Speaker's Bureau: Boehringer Ingelheim, Genomic Health, Roche, Pfizer; Shares: OncoMark Ltd; Travel and accommodation expenses: Pfizer, MSD, Abbvie, Astrazeneca, Novartis.

## Appendix Supplementary materials

Image, application 1

Image, application 2

Image, application 3

Image, application 4
