# Supplementary material for: Targeting mutant p53 with arsenic trioxide: A preclinical study focusing on triple negative breast cancer
Source: Transl Oncol. 2024 Jun 12;46:102025. doi: 10.1016/j.tranon.2024.102025 (PMC11225897; doi:10.1016/j.tranon.2024.102025)
Supplement: Supplementary file 4 [file mmc4.docx]

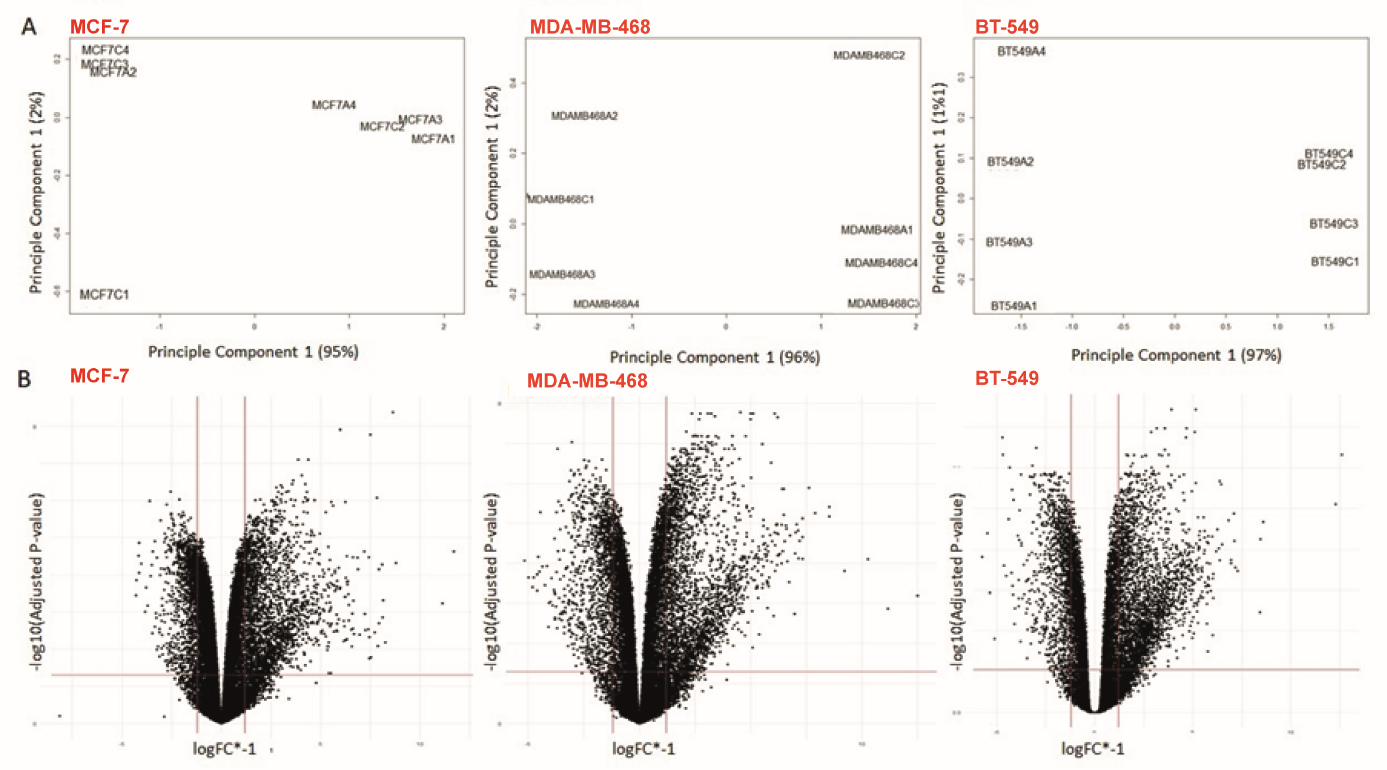


**Suppl. Fig. 1.** (A) Principal component analysis plot of the DEGs in MCF-7, MDA-MB-468 and BT-549 cells. (B) Volcanic plot showing the DEGs distribution in MCF-7, MDA-MB-468 and BT-549 cells.

**
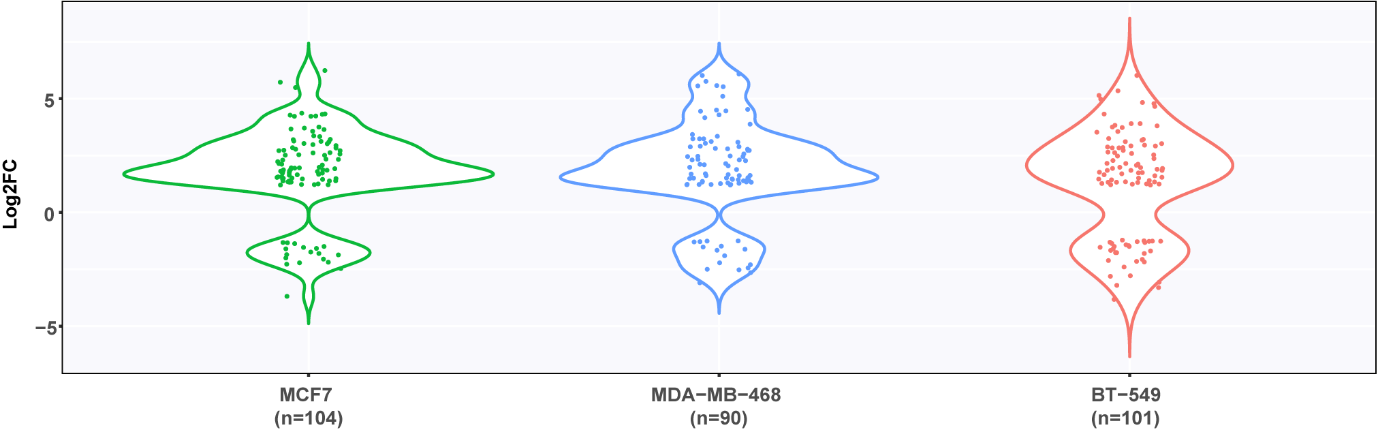
**

**Suppl. Fig. 2.** Violin plot showing log2 fold changes (Log2FC) in classical p53 target-gene expressions regulated by ATO in MCF-7 (Green), MDA-MB-468 (Blue) and BT-549 (Red) cell lines. 104 (MCF-7), 90 (MDA-MB-468) and 101 (BT-549) classical p53 target-genes are differentially regulated after treatment with 10 µM ATO for 8 h.

**Suppl. Table 1 : List of 20 cell lines used with their molecular subtype**^1^**, ER, PR and HER2 status**^1^**, p53 mutation present**^2^ **and the p53 mutational subtype**^3,4^**.**

| **Cell line** | **Molecular Subtype** | **ER** | **PR** | **ERBB2/**  **HER2** | **p53 Mutation** | **p53**  **Mut Subtype** |
| --- | --- | --- | --- | --- | --- | --- |
| BT-20 | TN | - | - | - | K132Q | Structural |
| BT-474 | Her2+ | + | + | + | E285K | Structural |
| BT-549 | TN | - | - | - | R249S | Structural |
| CAL-51 | TN | - | - | - | WT | WT |
| CAL-85-1 | TN | - | - | - | K132E | Structural |
| CAMA1 | Luminal | + | +/- | - | R280T | Contact |
| HCC1143 | TN | - | - | - | R248Q | Contact |
| HCC1937 | TN | - | - | - | R306* | Truncated |
| HCC70 | TN | - | - | - | R248Q | Contact |
| Hs578T | TN | - | - | - | V157F | Structural |
| JIMT1 | Her2+ | - | - | + | R248W | Contact |
| MCF-7 | Luminal | + | + | - | WT | WT |
| MDA-MB-231 | TN | - | - | - | R280K | Contact |
| MDA-MB-453 | TN | - | - | - |  | Deletion |
| MDA-MB-468 | TN | - | - | - | R273H | Contact |
| SKBR3 | Her2+ | - | - | + | R175H | Structural |
| SUM159 | TN | - | - | - | R158fsS | Insertion |
| T-47D | Luminal | + | + | - | L194F | Structural |
| UACC812 | Her2+ | + | +/- | + | WT | WT |
| ZR-75-1 | Luminal | + | +/- | - | WT | WT |

1. Dai X, Cheng H, Bai Z, Li J. Breast cancer cell line classification and its relevance with breast tumor subtyping. *J Cancer* 2017; **8**: 3131–3141.

2 The TP53 Website - The TP53 Web Site. https://p53.fr/ (accessed 28 Nov2023).

3 Joerger AC, Fersht AR. Structure-function-rescue: the diverse nature of common p53 cancer mutants. *Oncogene* 2007; **26**: 2226–2242.

4 Synnott NC, Murray A, McGowan PM, Kiely M, Kiely PA, O'Donovan N, O'Connor DP, Gallagher WM, Crown J, Duffy MJ. Mutant p53: a novel target for the treatment of patients with triple-negative breast cancer? Int J Cancer. 2017 Jan 1;140(1):234-246.

**Suppl. Table 2: Primer sequences used for RT-qPCR validation of p53-induced genes.**

| **Gene** | **Forward Primer** | **Reverse Primer** |
| --- | --- | --- |
| *ACTB* | ACAGAGCCTCGCCTTTGCC | GATATCATCATCCATGGTGAGCTGG |
| *BBC3* (PUMA) | GGATGAAATTTGGCATGGGGT | CCCTGGGGCCACAAATC |
| *CDKN1A* | TGCCGAAGTCAGTTCCTTGT | GTTCTGACATGGCGCCTCC |
| *HMOX1* | ACCTTCCCCAACATTGCCAG | CAACTCCTCAAAGAGCTGGATG |
| *MDM2* | AGGAGATTTGTTTGGCGTGC | TGAGTCCGATGATTCCTGCTG |
| *PMAIP1* (NOXA) | TGCAGGACTGTTCGTGTTCA | CTCGACTTCCAGCTCCGC |
| *PPM1D* (WIP1) | GTCCACACTCTTGACCCTCAG | ATTGTCCATGCTCACCCATC |
| *SESN2* | CCTTCCGGGCCCAGGATTATAC | GCTGGTTCACCTCCCCATAAT |
| *SFN*  (14-3-3-σ) | CCACTACGAGATCGCCAACA | CGTCCACAGTGTCAGGTTGT |
| *SLC7A11* | CTACTATGGTCAGAAAGCCTGT | CAGCATAAGACAAAGCTCCAAA |
| *SRXN1* | GGACACGATCCGGGAGGAC | TGCTCCCAGGTACACCCTTA |
| *TRIB3* | GAGACTCGCAGCGGAAGTG | GGGTGGCTCGCATCTCG |
| *TXNRD1* | CGATCTGCCCGTTGTGTTTG | ATGTTCCTCCGAGACCCCAT |

**Suppl. Table 3: Top 25 differentially regulated genes resulting from ATO treatment of MCF-7.**

|  | **GeneID** | **Symbol** | **logFC** | **adj.P.Val** |
| --- | --- | --- | --- | --- |
| 1 | 3162 | HMOX1 | 8.638746 | 4.29E-09 |
| 2 | 4502 | MT2A | 5.994566 | 1.31E-08 |
| 3 | 4501 | MT1X | 7.504001 | 1.75E-08 |
| 4 | 23657 | SLC7A11 | 4.377709 | 9.25E-08 |
| 5 | 29948 | OSGIN1 | 3.869314 | 9.25E-08 |
| 6 | 81631 | MAP1LC3B | 4.054833 | 2.41E-07 |
| 7 | 57761 | TRIB3 | 3.615982 | 3.44E-07 |
| 8 | 54541 | DDIT4 | 4.227106 | 4.22E-07 |
| 9 | 54550 | NECAB2 | 3.557618 | 4.25E-07 |
| 10 | 2730 | GCLM | 3.0013 | 4.98E-07 |
| 11 | 7296 | TXNRD1 | 3.086349 | 5.98E-07 |
| 12 | 2114 | ETS2 | 2.75745 | 5.98E-07 |
| 13 | 9181 | ARHGEF2 | 3.377069 | 6.85E-07 |
| 14 | 1843 | DUSP1 | 4.31054 | 7.16E-07 |
| 15 | 140809 | SRXN1 | 3.281169 | 7.16E-07 |
| 16 | 7026 | NR2F2 | -2.54042 | 7.16E-07 |
| 17 | 6509 | SLC1A4 | 2.870863 | 7.75E-07 |
| 18 | 8877 | SPHK1 | 3.054703 | 8.51E-07 |
| 19 | 4494 | MT1F | 7.842829 | 9.52E-07 |
| 20 | 1647 | GADD45A | 4.223081 | 9.52E-07 |
| 21 | 8795 | TNFRSF10B | 2.780626 | 9.95E-07 |
| 22 | 83667 | SESN2 | 4.361859 | 1.14E-06 |
| 23 | 54676 | GTPBP2 | 2.982901 | 1.18E-06 |
| 24 | 643246 | MAP1LC3B2 | 3.501505 | 1.18E-06 |
| 25 | 10221 | TRIB1 | 2.987761 | 1.26E-06 |

**Suppl. Table 4: Top 25 differentially regulated genes resulting from ATO treatment of MDA-MB-468.**

|  | **GeneID** | **Symbol** | **logFC** | **adj.P.Val** |
| --- | --- | --- | --- | --- |
| 1 | 5366 | PMAIP1 | 4.530335 | 1.89E-08 |
| 2 | 7422 | VEGFA | 3.234636 | 2.29E-08 |
| 3 | 23645 | PPP1R15A | 4.992362 | 2.29E-08 |
| 4 | 83667 | SESN2 | 5.105542 | 2.29E-08 |
| 5 | 7296 | TXNRD1 | 2.731313 | 2.29E-08 |
| 6 | 467 | ATF3 | 6.088614 | 2.29E-08 |
| 7 | 81631 | MAP1LC3B | 3.065878 | 2.29E-08 |
| 8 | 8140 | SLC7A5 | 2.365337 | 2.29E-08 |
| 9 | 26136 | TES | 3.214985 | 2.29E-08 |
| 10 | 23657 | SLC7A11 | 6.225249 | 2.38E-08 |
| 11 | 1604 | CD55 | 3.386645 | 2.38E-08 |
| 12 | 1491 | CTH | 4.991239 | 3.01E-08 |
| 13 | 6520 | SLC3A2 | 2.671671 | 3.49E-08 |
| 14 | 8878 | SQSTM1 | 2.111893 | 8.65E-08 |
| 15 | 84085 | FBXO30 | 3.439608 | 8.65E-08 |
| 16 | 55122 | AKIRIN2 | 2.492287 | 8.65E-08 |
| 17 | 441172 | FLJ46906 | 4.778761 | 8.65E-08 |
| 18 | 84803 | AGPAT9 | 4.191198 | 8.65E-08 |
| 19 | 2617 | GARS | 2.455491 | 8.65E-08 |
| 20 | 7779 | SLC30A1 | 4.161799 | 8.65E-08 |
| 21 | 83931 | STK40 | 2.566834 | 8.65E-08 |
| 22 | 80315 | CPEB4 | 2.79633 | 8.65E-08 |
| 23 | 2730 | GCLM | 3.094685 | 8.65E-08 |
| 24 | 6509 | SLC1A4 | 2.619873 | 8.65E-08 |
| 25 | 6809 | STX3 | 2.414338 | 8.86E-08 |

**Suppl. Table 5: Top 25 differentially regulated genes resulting from ATO treatment of BT-549.**

|  | **GeneID** | **Symbol** | **logFC** | **adj.P.Val** |
| --- | --- | --- | --- | --- |
| 1 | 83667 | SESN2 | 5.144272 | 5.12E-10 |
| 2 | 80315 | CPEB4 | 3.89983 | 5.12E-10 |
| 3 | 23710 | GABARAPL1 | 4.672873 | 1.97E-09 |
| 4 | 57761 | TRIB3 | 3.582065 | 1.97E-09 |
| 5 | 7316 | UBC | 3.158163 | 1.97E-09 |
| 6 | 90637 | ZFAND2A | 5.096419 | 2.54E-09 |
| 7 | 2805 | GOT1 | 2.875132 | 2.54E-09 |
| 8 | 9518 | GDF15 | 4.656103 | 3.68E-09 |
| 9 | 7057 | THBS1 | -4.6943 | 3.68E-09 |
| 10 | 11054 | OGFR | -2.99069 | 7.83E-09 |
| 11 | 80853 | KDM7A | 3.573439 | 8.86E-09 |
| 12 | 3310 | HSPA6 | 12.59964 | 1.26E-08 |
| 13 | 23657 | SLC7A11 | 3.766492 | 1.26E-08 |
| 14 | 9052 | GPRC5A | 3.279057 | 1.26E-08 |
| 15 | 5621 | PRNP | 1.607898 | 1.26E-08 |
| 16 | 3491 | CYR61 | -4.66935 | 1.26E-08 |
| 17 | 2617 | GARS | 2.008042 | 1.30E-08 |
| 18 | 54800 | KLHL24 | 3.434524 | 1.32E-08 |
| 19 | 7726 | TRIM26 | 2.604604 | 1.32E-08 |
| 20 | 64412 | GZF1 | 2.583881 | 1.32E-08 |
| 21 | 51646 | YPEL5 | 1.688436 | 1.32E-08 |
| 22 | 81631 | MAP1LC3B | 2.425909 | 1.45E-08 |
| 23 | 65117 | RSRC2 | 1.937697 | 2.30E-08 |
| 24 | 2535 | FZD2 | -3.17477 | 2.71E-08 |
| 25 | 51278 | IER5 | 3.727766 | 2.89E-08 |

**Suppl. Table 6: Thirty-three differentially regulated classical p53 target-genes in all three cell lines- MCF-7, MDA-MB-468 and BT-549.**

|  | **GeneID** | **Symbol** |
| --- | --- | --- |
| 1 | 63874 | ABHD4 |
| 2 | 25841 | ABTB2 |
| 3 | 23237 | ARC |
| 4 | 467 | ATF3 |
| 5 | 27113 | BBC3 |
| 6 | 694 | BTG1 |
| 7 | 79714 | CCDC51 |
| 8 | 1026 | CDKN1A |
| 9 | 113189 | CHST14 |
| 10 | 80315 | CPEB4 |
| 11 | 1847 | DUSP5 |
| 12 | 9518 | GDF15 |
| 13 | 54626 | HES2 |
| 14 | 22824 | HSPA4L |
| 15 | 51278 | IER5 |
| 16 | 26471 | NUPR1 |
| 17 | 5228 | PGF |
| 18 | 257068 | PLCXD2 |
| 19 | 5366 | PMAIP1 |
| 20 | 5900 | RALGDS |
| 21 | 127544 | RNF19B |
| 22 | 6301 | SARS |
| 23 | 5268 | SERPINB5 |
| 24 | 83667 | SESN2 |
| 25 | 7779 | SLC30A1 |
| 26 | 6548 | SLC9A1 |
| 27 | 9263 | STK17A |
| 28 | 8795 | TNFRSF10B |
| 29 | 8793 | TNFRSF10D |
| 30 | 205860 | TRIML2 |
| 31 | 80705 | TSGA10 |
| 32 | 219699 | UNC5B |
| 33 | 118738 | ZNF488 |
